# NETs and the thrombo-immune microenvironment: immune heterogeneity in recanalization, reperfusion, hemorrhagic transformation, and antithrombotic strategies

**DOI:** 10.3389/fneur.2026.1918580

**Published:** 2026-09-28

**Authors:** Fangfang Zhu, Feng Wang, Shuhua Wang, Feng Wang, Feng Zhang

**Affiliations:** 1 Department of General Practice, Shandong Provincial Hospital Affiliated to Shandong First Medical University, Jinan, China; 2 Department of Pharmacy, Affiliated Hospital of Shandong Academy of Chinese Medical Sciences, Jinan, China; 3 Department of Rehabilitation, Jinan Maternal and Child Health Hospital, Jinan, China; 4 Department of Neurology, Shandong Second Provincial General Hospital, Jinan, China

**Keywords:** acute ischemic stroke, DNase I, hemorrhagic transformation, immunothrombosis, neutrophil extracellular traps, platelet activation, precision antithrombotic therapy, recanalization

## Abstract

Reperfusion therapies have revolutionized the treatment of acute ischemic stroke (AIS), but target-artery recanalization, downstream angiographic reperfusion, microvascular tissue reperfusion, and functional recovery are distinct endpoints. Patients may have persistent occlusion or early reocclusion, incomplete modified Thrombolysis in Cerebral Infarction (mTICI) reperfusion, microvascular no-reflow despite high-grade mTICI/eTICI reperfusion, hemorrhagic transformation (HT), or poor 90-day functional outcome. Neutrophil extracellular traps (NETs) form a mechanistic link between thrombus structure, innate immune activation, endothelial injury, and antithrombotic responses. NETs provide a DNA-histone scaffold that stabilizes fibrin-rich thrombi, recruits platelets and coagulation factors, and exposes the neurovascular unit to cytotoxic granular proteins. The biological characteristics of NETs nevertheless vary among patients. Differences in neutrophil priming, platelet activation, monocyte-endothelial signaling, thrombus composition, collateral status, concurrent inflammation, and reperfusion injury create distinct thrombo-immune microenvironments. This article reviews how NET-driven immunothrombosis affects arterial recanalization, angiographic and tissue reperfusion, HT, and emerging antithrombotic strategies. It also proposes an operational immune-phenotyping framework with candidate biomarkers, assay-dependent research thresholds, sampling time points, and trial-level decision rules.

## 1 Introduction

Intravenous thrombolysis and endovascular thrombectomy have reshaped the treatment of acute ischemic stroke, particularly for patients with large-vessel occlusion (1, 2). The relevant endpoints must, however, be named precisely. Recanalization denotes reopening or patency of the previously occluded target artery. By contrast, the mTICI and eTICI scales grade the extent of downstream angiographic reperfusion within the affected arterial territory; neither is simply a target-artery recanalization score (3, 4). Microvascular tissue reperfusion requires perfusion-sensitive imaging or physiological assessment, and functional outcome is commonly assessed with the modified Rankin Scale (mRS) at 90 days. These endpoints can dissociate: a patient may have target-vessel patency but incomplete angiographic reperfusion (3, 5), high-grade mTICI/eTICI reperfusion yet persistent microvascular no-reflow (6, 7), or high-grade angiographic reperfusion followed by HT or poor functional recovery (8, 9). Accordingly, this review reports arterial recanalization, angiographic reperfusion, tissue reperfusion, and clinical outcome as separate constructs rather than interchangeable labels.

These clinical gaps suggest that thrombus removal is only part of the problem. AIS thrombi are not passive fibrin emboli; they are biologically active structures that contain fibrin, platelets, erythrocytes, leukocytes, von Willebrand factor (vWF), extracellular DNA, histones, proteases, and inflammatory mediators (10, 11). Brinkmann et al. (12) first described NETs as antimicrobial chromatin structures. NETs have since been recognized as important participants in thrombosis and vascular inflammation (13). In AIS, NETs can stabilize fibrin, trap platelets, impair tissue plasminogen activator (tPA)-mediated thrombolysis, and promote endothelial injury (14, 15). From a clinical perspective, the key question is not just whether NETs exist, but why NET-driven thrombosis manifests differently among patients. Some occlusions are predominantly erythrocyte-rich and may respond more readily to lysis or retrieval, whereas fibrin-, platelet-, or NET-rich thrombi can be more resistant (16). This article considers NETs as regulators of a heterogeneous thrombo-immune microenvironment and discusses how this heterogeneity affects recanalization, reperfusion, HT, and future antithrombotic strategies. Current acute stroke protocols are based mainly on time from onset, imaging-defined tissue salvageability, and vascular status (1, 5). These factors remain essential, but they do not capture thrombus immune composition or downstream microvascular inflammation. Platelet- and NET-rich thrombi with a platelet-driven inflammatory phenotype may ultimately require different adjunctive therapies from erythrocyte-rich thrombi, providing a rationale for linking thrombus biology to treatment selection.

## 2 NETs as central regulators of the thrombo-immune microenvironment

Classical lytic NETosis involves chromatin decondensation, peptidylarginine deiminase 4 (PAD4)-mediated histone citrullination, nuclear-envelope rupture, and the release of DNA decorated with neutrophil elastase (NE), myeloperoxidase (MPO), cathepsin G, and histones (12, 17). In vascular inflammation, rapid non-lytic NET release can also occur, indicating that NET formation is a heterogeneous, context-dependent process (18). In AIS, multiple signals can prime neutrophils and increase their propensity to form NETs. Denorme et al. (19) identified platelets as a key source of HMGB1 in experimental ischemic stroke: platelet-specific HMGB1 deletion reduced plasma NET levels and infarct volume in mice, while neonatal NET-inhibitory factor (nNIF) protected male, female, diabetic, and aged mice and remained effective when administered 1 h after stroke onset. The breadth of this preclinical protection is noteworthy, although mouse-to-human translational limitations remain.

NETs, once released, become structural and biochemical regulators of thrombi. The negatively charged DNA backbone binds fibrin, vWF, fibronectin, platelets, and tissue factor-bearing microparticles, while histones and granule proteases provide procoagulant and cytotoxic signals (13, 20). Ducroux et al. (14) analyzed 108 AIS thrombi and found that NETs were concentrated in the outer layer of the thrombus; *in vitro*, DNase I accelerated tPA-induced thrombolysis, whereas DNase I alone was ineffective. Peña-Martínez et al. (21) further demonstrated in a mouse model that pharmacological modulation of NETs could reverse tPA resistance in thrombotic stroke. These findings suggest that NETs are not merely a histological phenomenon but can influence thrombolytic efficiency. The thrombo-immune microenvironment extends beyond NETs themselves. Activated platelets can bind neutrophils through P-selectin/PSGL-1, integrins, HMGB1, and TLR4-dependent signaling, forming a platelet-NET positive-feedback loop (19, 22). Zhou et al. (22) found that interactions between NETs and activated platelets enhanced procoagulant activity in AIS patients with internal carotid artery occlusion and were associated with poorer outcomes. Monocytes contribute tissue factor, cytokines, and inflammasome-related signaling (23, 24). Endothelial cells express adhesion molecules, release vWF, and become targets of histone and protease toxicity (24, 25). Oxidative stress and related inflammatory mediators can further alter thrombus structure (26). NETs are central to this network because they physically connect cellular and noncellular components, amplifying coagulation, inflammation, and endothelial damage.

Immune heterogeneity can develop at multiple levels. Systemic neutrophil states vary across health, infection, malignancy, and chronic inflammatory conditions (27). Thrombus origin is equally important: the proportions of platelets, fibrin, neutrophils, and extracellular DNA differ among cardioembolic, large-artery atherosclerotic, and cryptogenic thrombi (16, 28). Local vascular conditions, collateral flow, ischemia duration, and reperfusion patterns determine how NET-rich thrombi interact with the downstream microcirculation. This context dependence helps explain seemingly contradictory findings. NETs may be abundant in retrieved thrombi without consistently predicting procedural difficulty, because retrieval performance also depends on thrombus length, vascular anatomy, thrombus age, and erythrocyte content. Plasma cell-free DNA or MPO-DNA complexes may correlate with stroke severity, but their specificity may be limited because infection, malignancy, and other inflammatory conditions can also increase circulating NET-related markers (29, 30). Therefore, the core analytical unit should be the entire thrombo-immune microenvironment rather than an isolated NET marker. NETs are better understood as a convergence hub through which systemic inflammation, thrombus origin, and local vascular injury are translated into different procedural and neurovascular outcomes. This concept bridges mechanistic biology with clinical problems such as recanalization failure and HT (Figure 1). Comparable heterogeneity is also evident outside the cerebral circulation. In retrieved coronary thrombi from patients with ST-elevation myocardial infarction, NET burden showed considerable interindividual variation and was examined in relation to clinical and angiographic features, including thrombus burden, supporting the view that NET content modulates rather than dictates arterial thrombus behavior (31).

**Figure 1:**
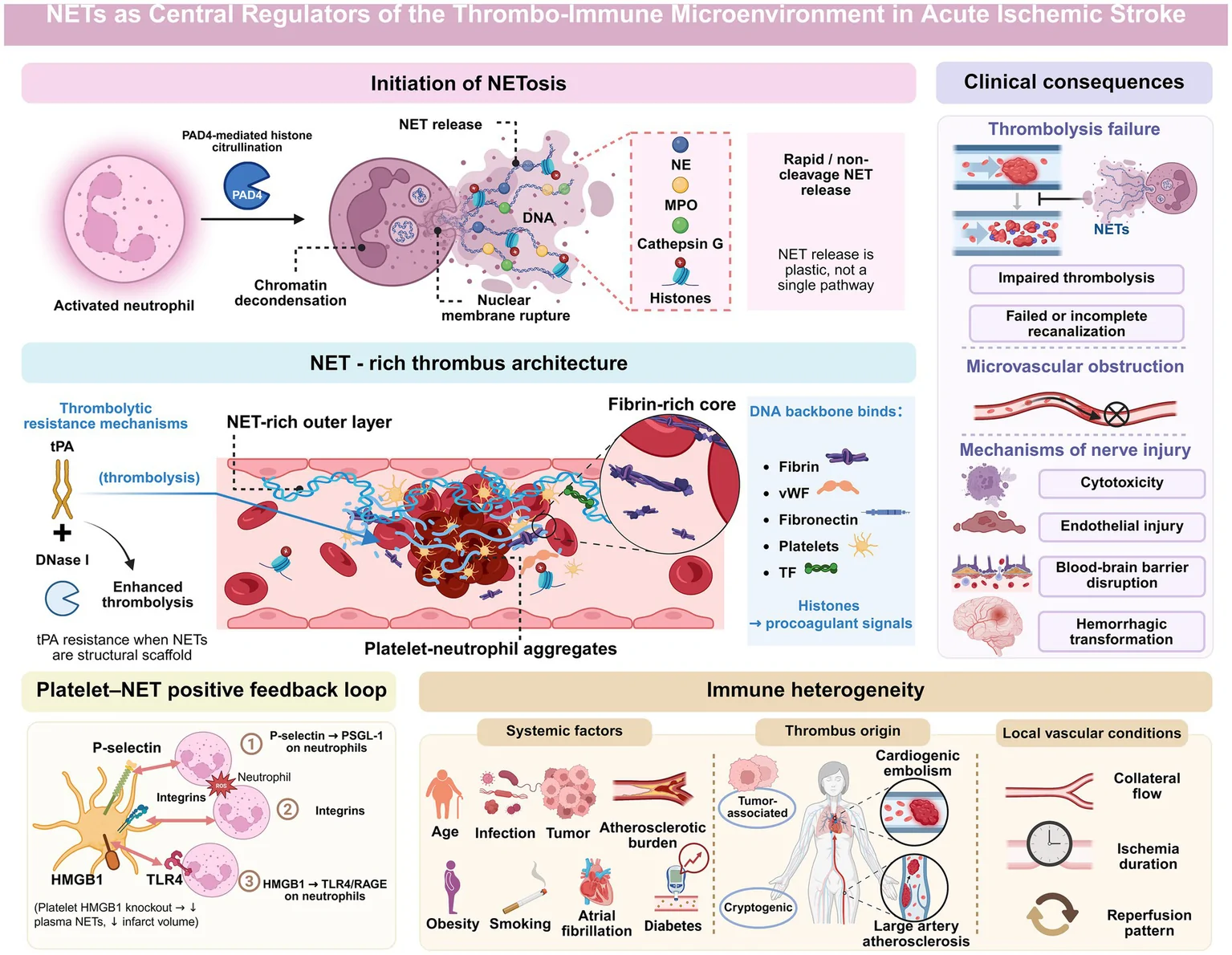
NET-driven thrombo-immune microenvironment in acute ischemic stroke.

## 3 Immune heterogeneity and clinical consequences

### 3.1 Arterial recanalization, angiographic reperfusion, and no-reflow

NET-rich thrombi may affect target-artery recanalization and downstream perfusion through distinct mechanisms. Extracellular DNA and histones increase thrombus density and elasticity, whereas NET-bound fibrin and vWF protect thrombi from tPA-mediated fibrinolysis (14, 20). Laridan et al. (15) documented NETs throughout retrieved AIS thrombi and found that adding DNase I to tPA improved ex vivo lysis. Novotny et al. (32) further found that thrombus NET content was associated with clinical outcome after thrombectomy; this association does not make the procedural and clinical endpoints equivalent.

Not every difficult-to-remove thrombus is NET driven. Erythrocyte-rich thrombi behave differently from platelet/fibrin-rich thrombi, and calcified or organized thrombi may resist retrieval for NET-independent reasons. Compared with simpler erythrocyte-rich thrombi, neutrophil- and platelet-rich thrombi may be more resistant to enzymatic breakdown, may fragment during thrombectomy, or may seed distal microthrombi. Circulating NET biomarkers also need not mirror the NET burden of the target thrombus because infection, malignancy, and systemic inflammation can independently increase plasma markers (30). Mechanistic attribution should therefore integrate target-vessel patency, the exact mTICI/eTICI grade, thrombus phenotype, blood biomarkers, imaging, and procedural behavior rather than infer ‘recanalization’ from a single measurement.

Mechanical thrombectomy further illustrates this distinction. Stent retrievers and aspiration catheters can reopen a proximal artery but may not clear distal emboli, capillary leukocyte plugs, or NET-rich microthrombi. NETs can trap erythrocytes and platelets within microvessels and thereby contribute to no-reflow. Thus, poor tissue reperfusion may persist despite target-vessel patency and high-grade angiographic reperfusion. Ng et al. (7) assessed no-reflow in patients with final eTICI 2c-3 using 24-h CT or MRI perfusion imaging. Studies should report target-artery recanalization or reocclusion and the final mTICI/eTICI grade with procedural variables (3, 4). Post-procedure tissue perfusion, infarct evolution, and 90-day mRS should be reported as separate outcomes (7, 8). The expression ‘successful reperfusion’ should be accompanied by the prespecified mTICI/eTICI threshold, and neither that label nor ‘recanalization’ should be used as a synonym for a good clinical outcome.

### 3.2 Hemorrhagic transformation

NET-rich immunothrombosis may also worsen neurovascular injury after macrovascular recanalization and downstream angiographic reperfusion. HT reflects vascular fragility, reperfusion pressure, infarct volume, BBB disruption, thrombolytic exposure, and inflammatory injury. NET formation can exacerbate BBB injury (33), and NET-associated histones are directly cytotoxic to endothelial cells (34). NET-associated DNA can activate cGAS-dependent innate immune signaling (33). Wang et al. (33) showed that tPA increased NET markers in ischemic brain tissue and plasma, that PAD4 was upregulated in tPA-treated ischemic brain tissue, and that DNase I treatment or PAD4 deficiency restored BBB integrity and reduced tPA-related bleeding through a cGAS-dependent mechanism. Separate preclinical work likewise links NET formation to ischemic brain damage (35). Rosell et al. (36) demonstrated that MMP-9-positive neutrophil infiltration during HT after human ischemic stroke was associated with BBB breakdown and type IV collagen degradation. Studies of HMGB1 deacetylation and cytokine-driven NET formation further support the view that NET biology is embedded in a broader inflammatory network (37, 38).

Immune heterogeneity may influence HT risk in ways that current algorithms do not capture. A patient with strong neutrophil activation, increased platelet–neutrophil aggregates, poor collaterals, and a large ischemic core may expose a vulnerable BBB to histones, proteases, and MMP activation when flow is restored. Another patient with a similar occlusion but less inflammatory priming may achieve reperfusion without bleeding. NETs may also interact with complement, endothelial adhesion pathways, and monocyte-derived cytokines to form different HT-risk phenotypes (24, 36). This heterogeneity helps explain why antithrombotic decisions after reperfusion therapy are difficult: bleeding risk depends not only on target-vessel status or drug exposure, but also on the immune state of the neurovascular unit. These data do not alter established eligibility for tPA or thrombectomy. Rather, they support prospective testing of NET-directed adjuncts within defined treatment windows and BBB states. Until such rules are validated, immune biomarkers should not by themselves delay reperfusion therapy or override guideline-based post-stroke antithrombotic timing.

## 4 Therapeutic opportunities targeting NET-driven immunothrombosis

### 4.1 Direct NET inhibition

The evidence above points to three separable clinical problems: resistance of a NET-rich target thrombus to lysis or retrieval, downstream microvascular NET obstruction despite macrovascular patency and angiographic reperfusion, and NET-driven BBB injury during reperfusion. These problems require different treatment windows and endpoints. DNase I directly dismantles the extracellular DNA scaffold. In Ducroux et al.’s (14) thrombus experiments, DNase I accelerated tPA-induced thrombolysis but was ineffective alone. Preclinical studies also link NET-targeted modulation to improved thrombolysis (21) and reduced BBB injury and hemorrhage (33). DNase I is nevertheless unlikely to dissolve a fibrin-rich thrombus by itself, and DNA degradation without adequate clearance may expose or leave bioactive histones and proteases. Timing, dose, local delivery, and combination with tPA or thrombectomy are therefore central translational questions. PAD4 inhibition acts upstream by limiting histone citrullination and chromatin decondensation. Denorme et al. (19) showed that GSK-199 pretreatment reduced plasma NET levels and infarct volume and improved neurological outcomes in mice; because it was administered before stroke onset, its post-onset therapeutic window remains unknown. PAD4-independent NET-like release and neuronal PAD4 expression also raise specificity and safety concerns. nNIF inhibits NET formation through a non-PAD4 mechanism, protected male, female, diabetic, and aged mice, and remained effective when administered 1 h after stroke onset (19). A realistic translational opportunity is short-term, phenotype-enriched adjunctive treatment during thrombolysis or thrombectomy, not long-term inhibition in all patients. Before reperfusion, NET dismantling might facilitate target-artery recanalization; during or after macrovascular reperfusion, the relevant endpoints are distal tissue perfusion, BBB integrity, and toxic histone/protease clearance. Combining NET degradation with histone neutralization, protease control, or enhanced clearance may therefore be more rational than treating NET inhibition as a generic anti-inflammatory strategy.

### 4.2 Modulating the platelet-NET axis

Because platelets are potent partners in NET induction during acute stroke, antiplatelet therapy may affect NET biology beyond conventional inhibition of platelet aggregation. Aspirin reduces thromboxane-dependent platelet activation, while P2Y12 inhibitors such as ticagrelor suppress ADP-dependent platelet signaling. Large clinical trials have tested ticagrelor-based antiplatelet strategies in acute ischemic stroke and TIA (39). In THALES, ticagrelor plus aspirin reduced the 30-day risk of stroke or death in patients with noncardioembolic, nonsevere ischemic stroke or high-risk TIA, but increased severe bleeding (40). Whether this benefit partly reflects reduced platelet-driven NET release has not been formally tested; the HMGB1-platelet-NET axis makes the hypothesis biologically plausible (19). In AIS, especially after thrombolysis, immediate intensification of antiplatelet therapy must be weighed against HT risk; the platelet-NET axis therefore requires phenotype-based testing rather than automatic treatment escalation. Other anti-inflammatory and antithrombotic strategies also warrant attention. Colchicine has anti-inflammatory effects and has been studied for plaque stabilization after acute coronary syndrome, but the cited study did not assess NET biology or AIS (41). HMGB1-TLR4 blockade and histone neutralization are mechanistically attractive (19, 34). Neutrophil-targeted delivery platforms and NLRP3 modulation remain preclinical (42, 43), as does factor XII targeting in cerebral ischemia (44). The common theme is not merely reducing thrombus formation but reshaping a thrombo-immune network that connects thrombus stability, microvascular occlusion, and BBB damage (Table 1).

**Table 1:** NET-targeted therapeutic opportunities in acute ischemic stroke.

Strategy	Main target	Potential benefit	Key translational concern
DNase I	Extracellular DNA scaffold	Improves lysis of NET-rich thrombi; may reduce microvascular obstruction	Insufficient for fibrin-rich clots alone; timing, histone/protease release, and combination with tPA need careful design
PAD4 inhibitors (e.g., GSK-199)	Histone citrullination / NET formation	Limits upstream NET generation; GSK-199 pretreatment reduced plasma NET levels and ischemic brain injury in mice	GSK-199 was administered before stroke onset; a post-onset treatment window is unestablished. PAD4-independent release, neuronal PAD4, and infection risk require study
Antiplatelet modulation (P2Y12, aspirin)	Platelet–neutrophil activation axis	May reduce platelet-driven NET release and recurrent immunothrombosis	Must balance early antiplatelet intensity against hemorrhagic transformation risk, especially after tPA
Colchicine	Neutrophil activation / inflammasome	May reduce inflammatory priming; a NET-specific effect in AIS remains unproven	Evidence in AIS remains indirect; systemic toxicity and infection risk need monitoring
Neonatal NET-inhibitory factor (nNIF)	NET formation (non-PAD4)	Non-PAD4 NET inhibition; effective when given 1 h after stroke onset in mice, including diabetic and aged models	Preclinical only; dosing, delivery route, durability, and human pharmacology remain undefined
Precision antithrombotic profiling	NET biomarkers, thrombus histology, BBB imaging	Matches adjunct therapy to NET-high or BBB-fragile/high-HT-risk phenotypes	Requires rapid, assay-specific cutoffs fitting the acute stroke workflow; prospective validation needed

### 4.3 Toward precision antithrombotic therapy

Precision strategies require a prespecified, time-resolved thrombo-immunophenotype rather than a retrospective list of markers. Candidate circulating measures include plasma or serum MPO-DNA complexes, citrullinated histone H3 (CitH3), cell-free DNA, neutrophil-to-lymphocyte ratio, and platelet–neutrophil aggregates (30, 45). Complementary domains include quantitative NET/platelet/fibrin histology, thrombus imaging features, collateral status, and BBB permeability (10, 36). No NET assay currently has an externally validated cutoff that can direct routine AIS treatment. In one prospective IVT-only cohort, pre-thrombolysis serum CitH3 > 17.67 ng/mL and MPO-DNA > 79.72 ng/mL were associated with early neurological deterioration (46). These receiver-operating-characteristic cutoffs were derived with specific serum ELISA kits in a single cohort and should be treated as assay-specific research thresholds, not universal clinical limits.

Prospective studies should use fixed collection points: before IVT or EVT without delaying treatment; during EVT and at final angiography; early after the procedure when tissue perfusion is clinically assessed; and at 24 ± 6 h for repeat biomarkers, NIHSS, and imaging. Day 3–7 sampling is optional for trajectory analysis. ‘NET-high’ should be defined prospectively using assay- and matrix-specific thresholds and, ideally, a two-domain rule that combines a NET-specific analyte with a second NET, platelet, or thrombus-histology domain. Active infection or malignancy and isolated cell-free DNA or NLR elevations should be classified as indeterminate.

These measurements are intended only for trial enrichment. Immune testing must not delay guideline-indicated IVT or EVT, and phenotypes should be matched to distinct trial endpoints: persistent target occlusion, incomplete mTICI/eTICI reperfusion, residual tissue-perfusion deficit despite patency and high-grade angiographic reperfusion, or BBB fragility/HT risk. The no-reflow definition used by Ng et al. (7) (final eTICI 2c-3 followed by 24-h perfusion imaging) provides a reference model, whereas earlier 0-6-h criteria require validation. Large-core measures should be recorded as HT-risk surrogates rather than direct BBB measures, and no biomarker threshold should change routine care until treatment utility is confirmed in biomarker-by-treatment interaction analyses. Table 2 provides the operational summary.

**Table 2:** Proposed operational immune-stratification framework for prospective validation.

Stage	Minimum measures	Primary endpoint/interpretation	Guardrail
Before IVT/EVT	CitH3 and/or MPO-DNA; platelet–neutrophil aggregates; cfDNA/NLR; infection/malignancy screen	Prespecified assay-specific NET-high definition; isolated cfDNA/NLR rise is indeterminate	Trial enrichment only; do not delay or alter IVT/EVT
During EVT and final angiogram	Target-artery patency; exact mTICI/eTICI grade; passes/distal embolization; quantitative thrombus histology	Report recanalization and angiographic reperfusion separately; assign phenotype only with prespecified cutoffs	NET-directed rescue only in trials
Early post-EVT (0–6 h exploratory; 24 ± 6 h reference)	Perfusion imaging when indicated; infarct evolution; repeat NET markers and NIHSS; BBB/HT imaging	No-reflow requires patency, high-grade mTICI/eTICI reperfusion, and residual tissue-perfusion deficit; BBB fragility is a separate phenotype	Do not change antithrombotic care from biomarkers alone
Day 3–7 and 90 days	Optional biomarkers; infarct volume; HT; 90-day mRS	Assess trajectory and biomarker-by-treatment interaction; analyze mRS separately from procedural endpoints	No bedside cutoff without external validation

## 5 Conclusion

In AIS, NETs connect coagulation, platelet activation, innate immunity, and endothelial injury. Their importance lies not only in their presence within a thrombus, but also in their capacity to define heterogeneous thrombo-immune microenvironments that may influence target-artery recanalization, downstream angiographic reperfusion, microvascular no-reflow, HT, and response to antithrombotic therapy. These outcomes should be measured separately: arterial patency or reocclusion, exact mTICI/eTICI grade, tissue-perfusion imaging and infarct evolution, HT, and 90-day mRS. Prospective studies should pair these endpoints with thrombus composition and serial circulating immune biomarkers. NET-targeted therapy is most likely to succeed when matched to a prespecified immunophenotype and treatment window and evaluated with endpoint-specific analyses rather than a composite notion of ‘successful recanalization.’

The immediate priority is therefore to validate an operational immune stratification framework. Baseline sampling before reperfusion therapy, intra-procedural angiographic and thrombus characterization, exploratory early tissue-perfusion assessment, and 24-h biomarker/imaging reassessment provide clinically meaningful time points. Assay-specific thresholds and the proposed two-domain rule can be used for prospective trial enrichment, but not yet for routine treatment selection. Patients with NET-rich, platelet-driven thrombosis and no prespecified BBB-fragility finding may be candidates for trials of adjunctive NET dismantling or platelet-NET modulation; patients with a BBB-fragile/high-HT-risk phenotype may be candidates for HT-prevention trials, while routine antithrombotic timing should continue to follow standard safety criteria. In this sense, NETs are less a single universal drug target than an entry point to precision reperfusion medicine.
